# AP2B1, a protein involved in endocytic trafficking, is associated with congenital heart defects when mutated

**DOI:** 10.1016/j.gendis.2026.102035

**Published:** 2026-01-10

**Authors:** Carla Guzman, WenJuan Zhu, Anupma Jha, Yanji Tong, Angelo Arrigo, Ivet Bahar, Cecilia Lo, Jiuann-Huey Ivy Lin

**Affiliations:** a Department of Critical Care Medicine, University of Pittsburgh School of Medicine, Pittsburgh, PA 15224, USA; b Centre for Cardiovascular Genomics and Medicine, Chinese University of Hong Kong, Hong Kong 999077, China; c Department of Pediatrics, University of Pittsburgh School of Medicine, Pittsburgh, PA 15201, USA; d Department of Biological Sciences, University of Pittsburgh, Pittsburgh, PA 15260, USA; e Department of Computational and Systems Biology, University of Pittsburgh School of Medicine, Pittsburgh, PA 15260, USA

Effective clinical management of congenital heart defects (CHDs) necessitates knowledge of their pathomechanisms. Our large-scale mouse forward genetic screen with N-ethyl-N-nitrosourea (ENU) mutagenesis revealed ten proteins in the endocytic trafficking pathway in CHD pathogenesis, including a missense mutation in *Ap2b1* (adaptor-related protein complex 2 subunit beta 1), a clathrin assembly protein complex member. Mice harboring a homozygous *Ap2b1* mutation (M448K) had the cardiac phenotypes of double outlet right ventricle and ventricular septal defects. Molecular dynamics simulations for the wild-type and mutant (M448K) proteins demonstrated molecular dynamics displacements close to zero in the M448K mutant protein of AP2B1 and its sequential neighbor, suggesting this mutation is highly constrained, which may undermine the protein function.

The clinical relevance of these findings is indicated by the recovery of 3 unrelated human individuals harboring rare putative damaging variants in *AP2B1* with a minor allele frequency < 0.01% among 1922 CHD patients at the Pediatric Cardiac Genomics Consortium (PCGC) and the UPMC Children's Hospital of Pittsburgh (CHP). These findings suggest that AP2B1 plays a crucial role in cardiovascular development and mutations in this gene may lead to structural instability, contributing to CHD development.

A forward genetic screening with chemical mutagenesis by N-ethyl-N-nitrosourea (ENU) models uncovered the *Ap2b1*^1^ mutant line after screening more than 80,000 mouse embryos using *in-utero* cardiac ultrasound imaging. Adaptor-related protein complex 2 (AP2) is a heterotetrameric adaptor complex, the most abundant component of clathrin-coated vesicles. It plays a critical role in regulating coat stiffness to support the initiation of membrane curvature, the initial step in endocytosis.

Using fetal echocardiography, followed by verification of cardiac morphology using episcopic confocal microscopy (ECM) in the chest, we identified a mutant mouse line 2321 (MGI 1919020). Mice harboring an *Ap2b1*^*M448K*^ missense mutation (*Ap2b1*^*m/m*^) in chromosome 11 exhibited homozygous lethality, with only 21% (*n* = 3 out of 14) survival until adulthood with normal cardiac anatomy. We observed that 50% (*n* = 7 out of 14) of *Ap2b1*^*m/m*^ demonstrated outflow tract defects with side-by-side great arteries consisting of double outlet right ventricle (Fig. 1B–F), 14% (*n* = 2 out of 14) of *Ap2b1*^*m/m*^ mutants had double outlet right ventricle with atrioventricular septal defect (Fig. 1C). Other cardiac phenotypes of *Ap2b1*^*m/m*^ mutants included perimembranous ventricular septal defect (*n* = 2 out of 14) (Fig. 1D), muscular ventricular septal defect (*n* = 1 out of 14) (Fig. 1I), hypoplastic transverse aortic arch (*n* = 2 out of 14 ), and right aortic arch (*n* = 2 out of 14) (Fig. 1H1, 1H2; Table S2). Most *Ap2b1*^*m/m*^ mutants had normal situs (93%, *n* = 13 out of 14) (Fig. S1D–S1F), and only one *Ap2b1*^*m/m*^ mutant with a cardiac phenotype of double outlet right ventricle and atrioventricular septal defect had an abnormal situs/heterotaxy (7%, 1 out of 14) with bilateral bi-lobed lungs (Fig. S1D', S1E') and dual inferior vena cava (IVC) (Fig. S1F'). However, the *Ap2b1*^*m/m*^ mutant with a heterotaxy phenotype has a normal 9 + 2 cilia structure in the airway using an electron microscope (Fig. S1G', S1H'). Whole-exome sequencing analysis demonstrated that the 2321 mutant mouse line resulted from an *Ap2b1* missense mutation (M448K) in the adaptin N-terminal region (Fig. 1N). The amino acid methionine in the 448 position is conserved among species (Fig. 1N). In addition to the cardiac phenotypes, *Ap2b1*^*m/m*^ mutants had extracardiac anomalies with craniofacial defects and micrognathia (*n* = 3 out of 14 *Ap2b1*^*m/m*^ mutants were examined) (Fig. S1A'), including the *Ap2b1*^*m/m*^ with heterotaxy. The *Ap2b1*^*m/m*^ mutant with heterotaxy also had a cleft palate and hypoplastic thymus. Consistent with the craniofacial phenotype in *Ap2b1* global knockout mice^2^ and pathogenic variants in humans,^3^ we observed that 2 *Ap2b1*^*m/m*^ mutants had cleft palate (2 out of 14 *Ap2b1*^*m/m*^ mutants) (Fig. S1B'). Furthermore, the normal thymus covered the proximal part of the great vessels (Fig. S1C), but in three out of 14 *Ap2b1*^*m/m*^ mutants that were examined, the hypoplastic thymus was noted with a visible proximal main pulmonary artery (Fig. S1C'). Both mutants with hypoplastic thymus had micrognathia. The details of cardiac and extracardiac phenotypes in *Ap2b1*^*m/m*^ mutants are listed in Tables S2 and S3.

**Fig. 1:**
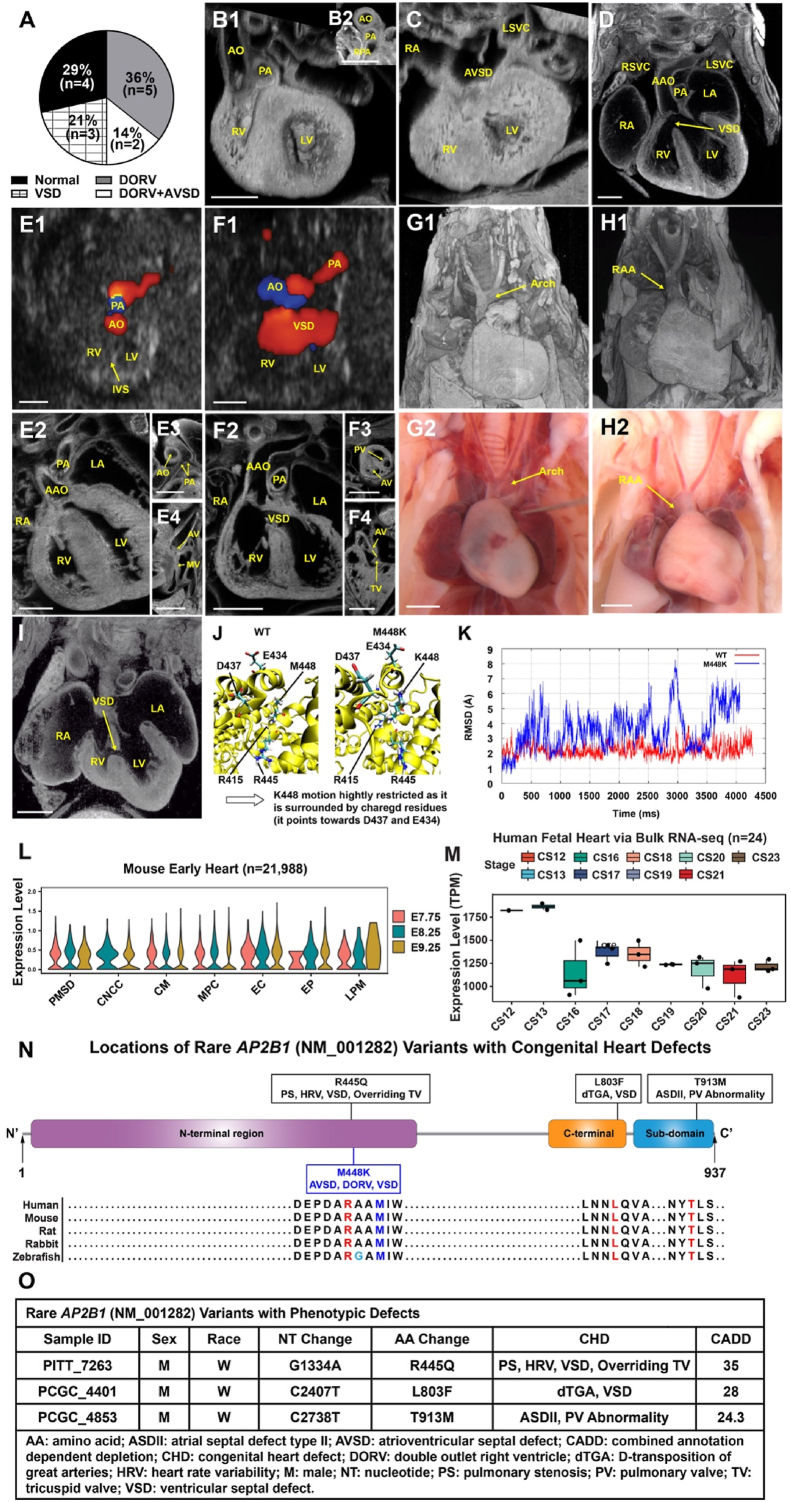
**(A**–**I)** Cardiac phenotypes of *Ap2b1*^*m/m*^ mutants. (A) The pie chart illustrates the percentage and cardiac phenotypes of *Ap2b1*^*m/m*^ mutants. Representative images of echocardiogram **(E1)** and episcopic confocal microscopy (ECM) in the control demonstrated an intact ventricular septum **(E1, E2)**, with the pulmonary artery (PA) located to the left and anterior to the aortic artery (AO) **(E3, E4)** showing aortomitral continuity. Vevo2100 color flow imaging of *Ap2b1*^*m/m*^ homozygous mutant demonstrated a ventricular septal defect (VSD), and both the aorta (AO) and pulmonary artery (PA) arise from the right ventricle (RV) **(F)**, with the aorta positioned to the right of the pulmonary artery, which was confirmed by ECM (B, F). Compared with a normal relationship between the aorta and pulmonary **(E3)**, the aorta is either side-by-side to the pulmonary valve **(F3)** or anterior to the pulmonary valve in the *Ap2b1*^*m/m*^ homozygous mutant **(B2)**. *Ap2b1*^*m/m*^ homozygous mutant lost the aorto-mitral continuity with a gap (yellow line) between the aortic and tricuspid valves **(F4)**. Representative 3D reconstruction **(G1)** and necropsy **(G2)** of a normal control demonstrated a normal aorta. Representative 3D reconstruction **(H1)** and necropsy **(H2)** of *Ap2b1*^*m/m*^ mutant demonstrate right aortic arch (RAA) (arrow). In addition to double outlet right ventricle, two *Ap2b1*^*m/m*^ mutants demonstrated atrioventricular septal defect (AVSD) (C). Three *Ap2b1*^*m/m*^ mutants had a cardio-phenotype of either peri-membranous VSD (D) or muscular VSD (I). **(J, K)** Structural and dynamic modularity of AP2B1 and effects of mutations M448K (AP2B1). (J) Ribbon diagrams displayed the dynamically coupled modules. Residues at hinge sites (crossover between oppositely moving blocks) are labeled. K448 motion is highly restricted as charged residues surround it (it points towards D437 and E434). (K) RMSD from equilibrium structure observed in dynamic modularity simulations of wild-type and mutated proteins (labeled), indicating the destabilizing effect of mutations. **(L, M)***AP2B1* is expressed in the developing and adult human and mouse hearts. (L) Analysis of published single-cell RNA sequencing from E7.75 to E9.25 demonstrated *Ap2b1* is expressed in all important cell lineages during cardiac development (from Miao et al,^4^ 21,988 cells). (M) Bulk RNA sequencing in human fetal heart demonstrated *AP2B1* is expressed since Carnegie stage (CS) 12 and throughout the stage during the cardiac development to CS 23 (From VanOudenho et al,^5^*n* = 24). **(N)** Schematic diagram showing locations of rare *AP2B1* (NM_001282) variants associated with congenital heart defects. AP2B1 consists of 937 amino acids with an N-terminal adaptin domain, an adaptin C-terminal domain, and a beta2-adaptin C-terminal sub-domain. The arginine residue at position 445, the leucine residue at 803, and the threonine residue at position 913 are conserved among species. The mutant line 2321 (MGI 1919020) results from an *Ap2b1*(NM_027,915) missense mutation (M448K) in the adaptin N-terminal region. The methionine residue mutated at position 448 is conserved among species. **(O)** A summary of the rare variants with phenotypes found in congenital heart defects. Scale bars: 0.5 mm in Figure 1B–I and 2 mm in Figure 1G2, H2. AA, amino acid; AAO, ascending aorta; AO, aorta; AVSD, atrioventricular septal defect; CM, cardiomyocyte; CNCC, cardiac neural crest cell; CS12-23, Carnegie stages 12–23; EC, endocardium; EP, epicardium; IVS, interventricular septum; LA, left atrium; LPM, lateral plate mesoderm; LSVC, left superior vena cava; LV, left ventricle; MPC, multipotent progenitor; MV, mitral valve; PA, pulmonary artery; PMSD, paraxial mesoderm; RA, right atrium; RAA, right aortic arch; RSVC, right superior vena cava; RV, right ventricle; TV, tricuspid valve; VSD, ventricular septal defect.

AP2 complex comprises α, β2, μ2, and s2 subunits. The mutated residue β2 M448 in AP2B1 neighbors the binding site for the endocytic YXXΦ motif. When the AP2 structure is inactive or closed, the β2-subunit blocks the μ2-subunit YXXΦ-binding site. M448 in AP2B1 and its sequential neighbors are highly constrained, with displacements close to zero using Gaussian Network Model (GNM) analysis (Fig. 1J). The effect of the mutation on the stability of AP2 was further examined by molecular dynamics simulations (Fig. 1K). The root mean square deviation from the original structure, which provides a measure of structural stability, exhibits large fluctuations in the mutant (blue curve) compared with the respective wild-type (red curve). This observation is consistent with the changes in free energy, ΔF, induced upon single-point mutations. A value of ΔF above 1.0 kcal/mol typically corresponds to substantial destabilization, at least on a local scale. M448K mutation leads to an even more dramatic increase in free energy in AP2B1 (ΔF = 4.8 kcal/mol). Although unaltered protein expression levels of β2-adaptin and α-adaptin were observed in the hearts of wild-type and mutants (Fig. S2F and S2G), this mutation may undermine the protein function based on the GNM and molecular dynamics analysis.

To investigate the expression of *Ap2b1* in the developing heart, we analyzed the published transcriptome of mouse hearts and human hearts. We observed a wide-range expression of *Ap2b1* in developing mouse hearts, including cardiac neural crest cells, outflow tract, and atrioventricular cushions (Fig. 1L; Fig. S2A–S2D). *Ap2b1* was expressed in the cardiomyocytes (Fig. S2B), endothelial cluster cells, and epicardial cells since E7.75. Since E8.25, *Ap2b1* was highly expressed in the cardiac neural crest cells^4^ (Fig. 1L; Fig. S2A). The similar wide-range expression of AP2B1 was observed in the developing and adult human hearts (Fig. S3). Immunofluorescence confirmed the localization of AP-2 complexes in the developing mouse heart, marked by β2-adaptin, including the developing outflow tract, atrioventricular cushions, and developing ventricles (Fig. S2E).

To investigate the role of AP2B1 in human disease, we observed that *AP2B1* was expressed in the developing human fetal heart by analyzing published bulk RNA-sequencing data from 9 Carnegie stages (CSs) (*n* = 24)^5^ (Fig. 1M). Analysis of human exome data from the Exome Aggregation Consortium (ExAC) database of > 60,000 individuals with and without CHD further showed that a PLI score (indicating the likelihood that a gene is intolerant to a loss-of-function mutation) and Z score for missense mutation of *AP2B1* are 1 and 5.17, respectively, indicating that *AP2B1* is highly intolerant to missense and loss-of-function mutations.

We identified three unrelated human individuals harboring nonsynonymous, rare putative damaging variants in *AP2B1* (NM_001282) with a minor allele frequency < 0.01% (Fig. 1N and O). The putative damaging variants mapped along the protein sequence revealed the locations of missense mutations in different regions: R445Q is in the N-terminal region and is close to the M448K mutation we identified in the mouse forward mutagenesis. This variant is associated with a single-ventricle CHD consistent with a large muscular inlet ventricular septal defect, straddling tricuspid valve, infundibular and supra-valvar pulmonary stenosis, and hypoplastic right ventricle with a canal branch from the right coronary artery across the right ventricular outflow tract. A male individual with a d-transposition of the great artery is associated with an L803F variant; the variant is in the adaptin C-terminal domain. A patient with an atrial septal defect and a pulmonary valve dysplasia had a heterozygous T913M variant. T913 M is in the beta2-adaptin C-terminal sub-domain, a region that interacts with arrestin β1 (ARRB1). ARRB1 is a cofactor in the beta-adrenergic receptor kinase, playing a role in the desensitization of β-adrenergic receptors.

In summary, we observed that AP2B1 was expressed in developing and mature hearts. In addition to the reported perinatal lethality and cleft palate,^2^ we identified a mutation in *Ap2b1* related to various CHDs in a mouse model. We also found three rare damaging variants in patients with different CHDs. Our observation provides insights into the under-appreciated association of endocytic trafficking proteins and CHDs.

## CRediT authorship contribution statement

**Carla Guzman:** Writing – original draft, Methodology, Investigation, Formal analysis, Data curation. **WenJuan Zhu:** Writing – review & editing, Writing – original draft, Software, Methodology, Formal analysis, Data curation. **Anupma Jha:** Methodology, Investigation. **Yanji Tong:** Writing – review & editing, Visualization, Methodology, Investigation. **Angelo Arrigo:** Project administration, Methodology, Investigation. **Ivet Bahar:** Writing – original draft, Software, Methodology, Investigation. **Cecilia Lo:** Supervision, Project administration, Methodology, Conceptualization. **Jiuann-Huey Ivy Lin:** Writing – review & editing, Writing – original draft, Supervision, Project administration, Methodology, Investigation, Data curation, Conceptualization.

## Conflict of interests

All authors declared no competing interests.
