# Supplementary material for: AP2B1, a protein involved in endocytic trafficking, is associated with congenital heart defects when mutated
Source: Genes Dis. 2026 Jan 10;13(6):102035. doi: 10.1016/j.gendis.2026.102035 (PMC13355575; doi:10.1016/j.gendis.2026.102035)
Supplement: Multimedia component 1 [file mmc1.docx]

**Supplemental Materials.**

**Recovery of the *Ap2b1* Mutation and Mouse Breeding**

Mouse studies were conducted under an approved University of Pittsburgh Institutional Animal Care and Use of Committee protocol. Breeding for the mutagenesis screen was described previously^1^. We used male and female mice aged between 6 weeks and 6 months in a C57BL6/J background. Genomic DNA for line 2321 (MGI: 1919020) was harvested from a mutant embryo displaying complex congenital heart defects including double out right ventricle (DORV) , atrioventricular septal defect (AVSD), right aortic arch, hypoplastic pulmonary artery, and D-malposed great arteries, then sequences captured using Agilent SureSelect Mouse All Exon Kit V1 and sequencing using Illumina HiSeq 2000 with a goal of 50X as the minimum target-sequence coverage (BGI America). Sequences were aligned to the C57BL/6J mouse reference genome (mm9) and then analyzed using CLCBio Genomic Workbench and the Genome Analysis Toolkit (GATK) software. Annotation of sequence variants was performed using ANNOVAR (http://www.openbioinformatics.org/annovar) and then filtered against custom in-house scripts as well as dbSNP128 (https://www.ncbi.nlm.nih.gov/projects/SNP/snp_summary.cgi?build_id=128). Eight homozygous coding mutations were identified [Supplemental Table 1]. More than 14 generations of the breeding line 2321 have further validated the *Ap2b1* mutation as responsible for the displayed phenotypes.

| Supplemental Table 1.  Eight homozygous coding mutations were identified from line 2321. | | | | | |
| --- | --- | --- | --- | --- | --- |
| **Gene** | **Chromosome** | **Position** | **Reference Seq ID** | **Nucleotide change** | **Amino Acid change** |
| *Adora2b* | 11 | 2062641 | NM_007413 | c.T38A | p.L13Q |
| *Ap2b1* | 11 | 83154847 | NM_027915 | c.T1343A | p.M448K |
| *Kis1c* | 11 | 70541728 | NM_153103 | c.T2650c | p.S884P |
| *Nfkb1* | 3 | 135276960 | NM_008689 | c.C737T | p.S246F |
| *Rgs22* | 15 | 36034328 | NM_001195748 | c.T340C | p.C114R |
| *Slc37a3* | 16 | 39314591 | NM_028123 | c.T109A | p.S37T |
| *Synj1* | 16 | 90981192 | NM_001164483 | c.1068+2T>A |  |
| *Usp53* | 3 | 122636800 | NM_133857 | c.C3050T | p.T1017I |

**Ultrasound assessment of cardiac structure**

A fetal echocardiogram was performed with Vevo 2100 biomicroscopes with the 40 MHz transducer providing 30 µm axial X 75 µm lateral resolution, and a standard combination of 2-dimensional, color flow, and spectral Doppler imaging were used in our fetal ultrasound phenotyping pipeline^1,2^. We screened with fetal ultrasound imaging at E14.5-E15.5 after completing major cardiovascular developmental processes, including OFT and ventricular chamber septation and development. This ensures the mutations recovered would have more clinical relevance, as only conceptuses with a fully formed heart would be represented in the patient population of congenital heart diseases^2^.

**Episcopic confocal microscopy histopathology (ECM)**

Embryos or newborns identified with abnormal cardiac structure by ultrasound screening were harvested and embedded in paraffin for ECM. The two-dimensional (2D) histopathology and three-dimensional (3D) reconstruction imaging techniques are the gold standard for the validation and diagnosis of any congenital heart disease^2^.

**Genotype**

Genotyping was performed using GoTaqTM Hot Start Polymerase: Green Master Mix, 2X (Promega, PRM5122). PCRs were performed on SimpliAmpTM Thermal Cycler (Fisher Scientific, A24811). Primers Forward: 5’-CAGCTTGGAATCCCTTGTGT-3’

Reverse: 5’-CACCTTCTCAGCTCTTGTTCTG-3’

**Immunostaining**

Embryos were fixed in 4% Paraformaldehyde (Electron Microscopy Sciences, 15710) overnight. Cryosections were then incubated at 4^o^C overnight for a primary antibody with an anti-β2 adaptin antibody (Proteintech 15690-1-AP, 1:1000 dilution). The secondary antibodies (1:1000 dilution, Invitrogen Alexa Fluor-555, Goat anti-rabbit IgG #A-21428), DAPI (4',6-Diamidino-2-Phenylindole) (1:1000 dilution, Invitrogen 62248) were incubated at room temperature for an hour. Images were performed using confocal microscopy on a Leica DMI6000.

**Immunoblotting/Western Blotting**

Whole hearts harvested at E14.5 were lysed in RIPA lysis buffer (ThermoFisher#89900) with protease inhibitor (Pierce # A32961). Proteins were quantified using BCA (Pierce#23225). Samples with 50μg of total protein were boiled in SDS sample buffer. SDS-denatured samples were resolved on NuPage Bis-Tris 4 to 12% gels (Invitrogen NP0321). Gels were transferred to the PVDF membrane. Membranes were blocked in Odyssey blocking buffer (LI-COR# 927-60001) for 1 hour at room temperature. Blocked PVDF membranes were incubated with primary antibodies (α-adaptin, 1:1000 ThermoFisher MA3-061, β2-adaptin, 1:1000 proteintech#15690, GAPDH, 1:1000 Santa Cruz SC-32233) in 3% bovine serum albumin (BSA, Sigma #A2153) in TBST overnight. After washing in TBST, secondary antibody combinations were IRDye 800CW donkey anti-mouse IgG (1:10,000 Li-COR#926-32212) and IRDye 680RD donkey anti-rabbit IgG (1:10,000, LI-COR#926-68073) in 3% BSA in TBST for 1 hour in the dark. Immunoblots were washed 3 times in TBST. Immunoblots were scanned in IR Odyssey imager-classic (LI-COR).

**Computational methods**

Details of the GNM theory and applications are described in our earlier work^3^. In brief, a Kirchhoff matrix based on C^α^-atoms pairs located within 7.0Å describes the protein's collective fluctuations modeled as an elastic network with uniform force constants. The method allows for identifying soft modes intrinsically accessible to the quaternary structure and provides insights into structural elements mediating the structural dynamics.

Molecular Dynamics (MD) simulations for the wild-type and mutant (M448K) proteins were performed using the AMBER12 package^4-6^(GPU version of the pmemd program), with the Amber12SB force field and using the TIP3P water model. The protocol consisted of an initial minimization in explicit solvent, using 5,000 steepest descent and 5,000 conjugate gradient steps, to remove strong steric contacts, followed by a temperature and pressure equilibration of 2.5 ns, and a production run of 90 ns. The systems were kept at a temperature of 300 K, using Langevin dynamics with a collision frequency of 2 ps-1; the SHAKE algorithm was adopted to use a 2fs time step.

Several methods have been developed to estimate the consequence of mutation on protein stability, including CC/PBSA, EGAD, FoldX, Rosetta, and I-Mutant2.0. These methods generally yield results in qualitative agreement (on the stabilizing or destabilizing effect of mutations). At the same time, they differ in the absolute (quantitative) evaluation of free energy changes^7^. We adopted I-mutant2.0^8^ for performing serial calculations for the effect of M448K point mutation. I-mutant2.0 is a support vector machine web server developed to predict protein stability changes upon point mutations. Schreiber and coworkers showed that I-mutant2.0 predictions predict the tendency of the mutation toward stabilization/destabilization of the structure (i.e., the sign of the free energy change) with an accuracy rate of up to 80%^7^.

**Human Study Participants**

The Institutional Review Board approved all data access requests and human studies. We obtained written informed consent from all participants and/or parents of children. The personal identities of the study participants were encrypted and secured by approved guidelines and regulations. Whole-exome sequencing (WES) data from in-home CHD patients from UPMC Children’s Hospital of Pittsburgh (CHP), CHD patients from the Pediatric Cardiac Genomics Consortium (PCGC)^9^ were downloaded and analyzed. The University of Pittsburgh Center for Research Computing partly supported this research by providing computing resources.

**Bioinformatic analysis of whole exome sequencing** **data**

To recover rare predicted pathogenic variants in *AP2B1*, we analyzed the WES data previously described^10,11^. WES data of 1922 CHD cases from the PCGC (dbGaP phs001194.v2.p2)^9^ and CHP and 2602 controls from the Alzheimer’s Disease Sequencing Project (ADSP)^12^ (NG00067.v2) were analyzed. Putative damaging missense (D_Mis) was called likely damaging by MAF <0.01%, and at least 4 of 9 prediction algorithms (SIFT, Polyphen2_HDIV, LRT, MutationTaster, MutationAssessor, FATHMM, PROVEAN, MetaSVM, M_CAP). Fisher’s exact test was used to estimate the P-value and the odds ratio (OR) with 95% confidence intervals (CI).

**Bioinformatic analysis of Publicly Available Single-Cell RNA Sequencing Data and bulk RNA sequencing Data**

Publicly available raw single-cell RNA sequencing (scRNA-seq) data of human and mouse fetal and adult hearts were downloaded from the NCBI GEO database or the corresponding database mentioned in the published papers. Downstream analyses were performed as described previously^11^. Bulk RNA sequencing data of the fetal human heart from Carnegie stages 12 to 23 were downloaded from the NCBI GEO database under accession number GSE138799. Expression counts were transferred to transcript per million (TPM).


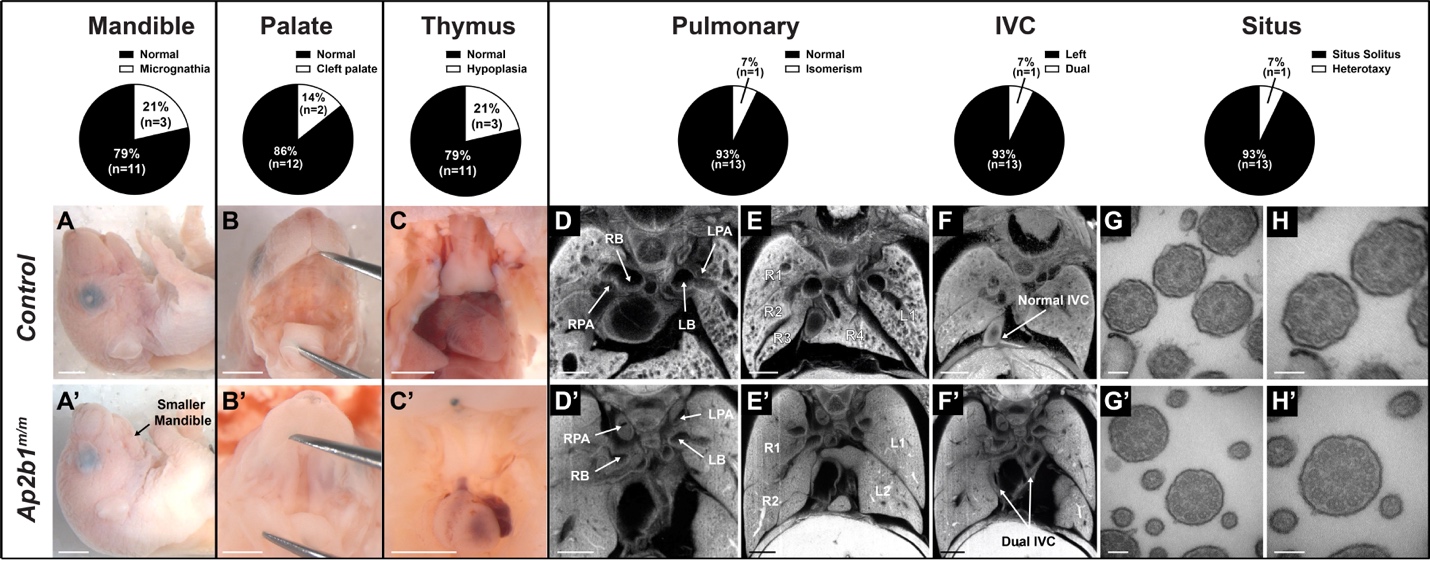


Supplemental Figure 1. Extracardiac phenotypes of *Ap2b1^m/m^* mutants. The pie chart illustrates the percentage of different extracardiac phenotypes of *Ap2b1^m/m^* mutant. Compared with the E15.5 control embryos (A), *the Ap2b1^m/m^* mutant had a short mandible (A’). (B) E15.5 controls demonstrated an intact palate, and the *Ap2b1^m/m^* mutant had a cleft palate with a gap between left and right palates (B’) and a hypoplastic thymus (C’). (D) Representative normal bronchial-arterial relationships in control; the left pulmonary artery (LPA) crosses above the left bronchus (LB), and the right pulmonary artery (RPA) crosses between the right bronchi (RB). (D’) Both pulmonary arteries cross above the bronchi in 2321-006-NMB, *Ap2b1^m/m^* mutant. (E) There are 4 lobes of the right lung and one lobe on the left lung in the normal control. (E’) 2321-006-NMB mutant had 2 lobes on the right and 2 lung lobes on the left. Instead of normal right-sided inferior vena cava (F), this 2321-006-NMB mutant also had dual inferior vena cava, one on the left side and one on the right side (F’). There is no difference in cilia morphology from the respiratory tract between *Ap2b1^m/m^* mutant with heterotaxy and its controls (G, G’, H, H’). Scale bars: 2mm (A-C, A’-C’), 0.5mm (D-F, D’-F’), EM: 100nm (G, G’, H, H’). IVC: inferior vena cava, L: left lobe, LB: left bronchus, LPA: left pulmonary artery, R: right lobe, RB: right bronchus, RPA: right pulmonary artery.


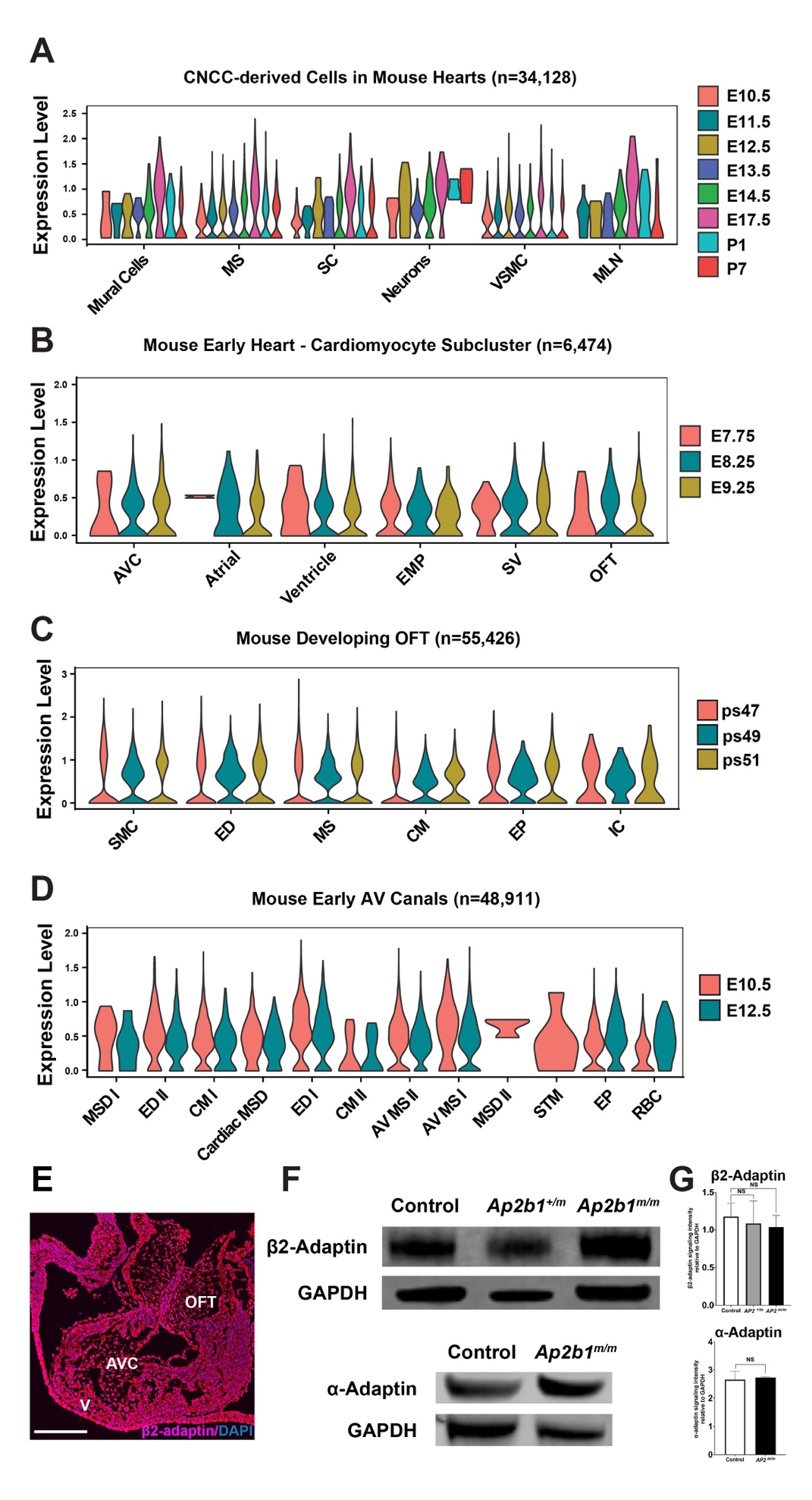


Supplemental Figure 2*. Ap2b1* is expressed in the developing mouse hearts (A). Analyzing different stages of CNCC-derived cells, we observed that Ap2b1 is expressed in various cell clusters, including mesenchymal cells (MSC) and vascular smooth muscle cells (VSMC) (Chen *et al.*^13^, 34,128 cells). (B) Further investigation into the cardiomyocyte subpopulation (from Miao *et al.*^14^, 6474 cells) showed that *Ap2b1* is expressed in the developing atrial cells, ventricular cells, cardiomyocytes in the outflow tract (OFT), and atrioventricular cushion (AVC) (C). Analyzing the RNA-seq from the 47-somite, 49-somite, and 51-somite-stage OFT, demonstrated *Ap2b1* is expressed in all the cell lineages (Liu *et al.*^15^, 55426 cells). (D) *Ap2b1* is expressed in all the cell lineages in the developing AVCs at E10.5 and E12.5 (from Lotto *et al.*^16^, 48911 cells). (E) Localization of *Ap2b1* protein in mouse hearts was confirmed by immunofluorescence, which is widely expressed in the developing OFT, AVC, and developing ventricle (V). (F) Immunoblotting of β2-adaptin antibody and α-adaptin demonstrated AP2B1 is expressed in the developing heart (E14.5) of wild-type and mutant embryos. GAPDH was used as the loading control. (G) Quantification of protein abundance of β2-adaptin and α-adaptin relative to GAPDH expression.

AVC: atrioventricular cushion, AVMS: atrioventricular mesenchyme, CM: cardiomyocyte, CNCC: cardiac neural crest cell, E: embryonic day, EC: endocardial cell, ED: endothelial cell, EMP: endothelial microparticles, EP: epicardial cell, GAPDH: Glyceraldehyde-3-phosphate dehydrogenase, IC: immune cell, LPM: lateral plate mesoderm, MPC: multipotent progenitor, MS: mesenchyme, MSD: mesoderm, NT: neural tube, OFT: outflow tract, P: postnatal day, PMSD: paraxial mesoderm, RBC: red blood cell, SC: Schwann cell, SMC: smooth muscle cell, STM: septum transversum mesenchyme, SV: sinus venosus, VSMC: vascular smooth muscle cell, MLN: melanocyte.


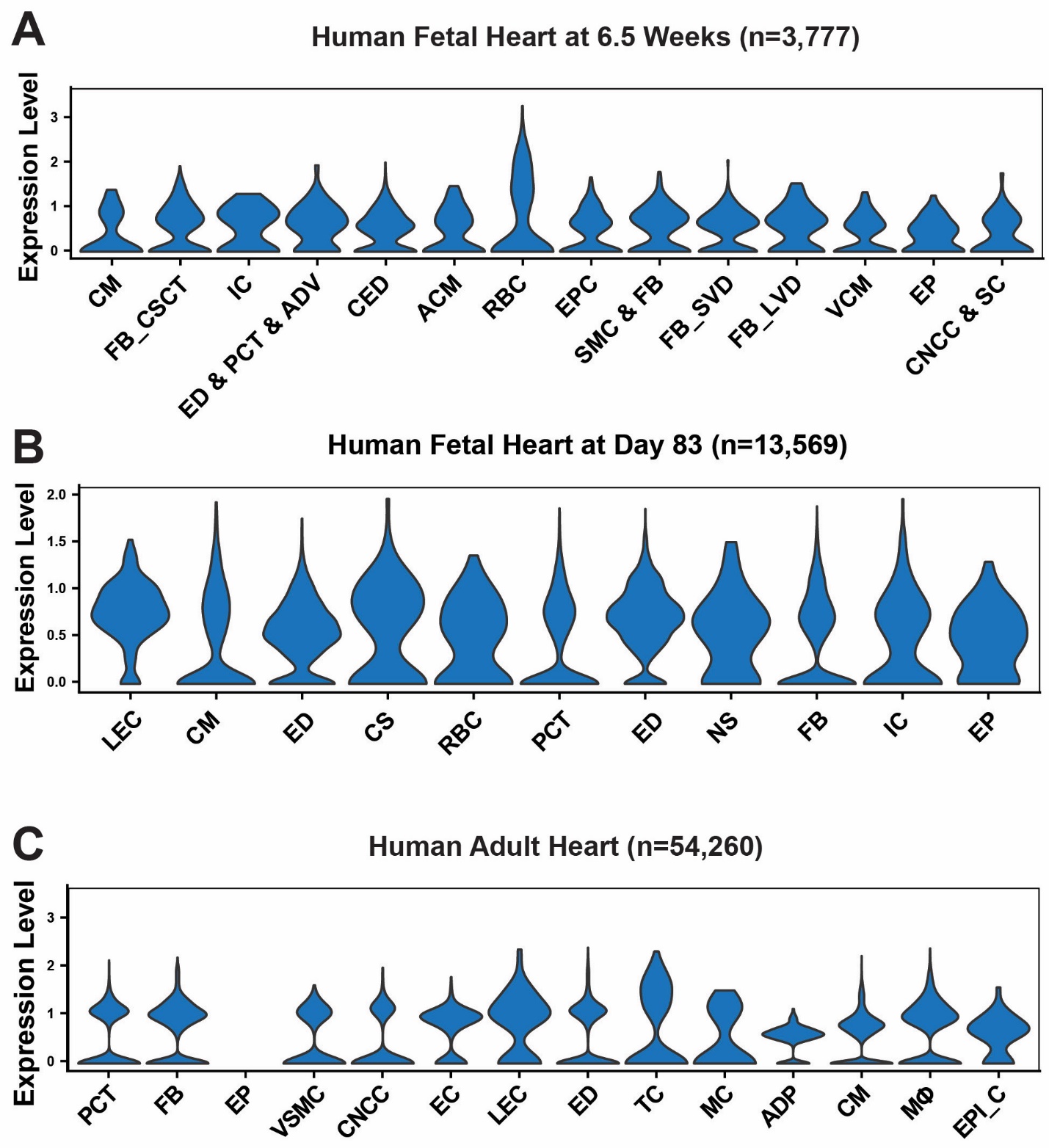


Supplemental Figure 3. *AP2B1* is expressed in the major cell types in the developing human heart as well as adult hearts. (A) analyzing single-cell scRNA-seq datasets in 6.5 weeks of human fetal hearts (from Asp *et al.*^17^,3,777 cells) and (B) later, at the gestational age of 83 days old fetal heart (from Miao *et al.*^14^,13,569 cells). *AP2B1* is broadly expressed in all the major cell lineages in the heart, including cardiomyocytes (CM), both atrial and ventricular cardiomyocytes (ACM, VCM), cardiac neural crest cells (CNCC), endothelial cells (ED), immune cells (IC), epicardial cells (EP), and mesenchymal and fibroblast cells (SMC and FB). (C) A*P2B1* is expressed in all the cell lineages in the adult human heart except epicardial cells, but *AP2B1* is expressed in the epicardial-derived cells (from Hill *et al*.^18^ 54,260 cells).

ACM: atrial cardiomyocyte, ADP: adipocyte, ADV: adventitia, CED: capillary endothelium, CM: cardiomyocyte, CNCC: cardiac neural crest cell, CS: conduction system cell, EC: endocardial cell, ED: endothelial cell, EP: epicardial cell, EPC: epicardial-derived cell, EPI_C: epithelial-like cell, FB: fibroblast, FB_CSCT: fibroblast-like cardiac skeleton connective tissue, FB_LVD: fibroblast-like larger vascular development, FB_SVD: fibroblast-like smaller vascular development, IC: immune cell, LEC: lymphatic endothelial cell, MC: mast cell, MΦ: macrophage, NS: nervous system cell, PCT: pericyte, RBC: red blood cell, SC: Schwann cell, SMC: smooth muscle cell, TC: T cell, TPM: transcripts per million, VCM: ventricular cardiomyocyte, VSMC: vascular smooth muscle cell.

| Supplemental Table 2. Cardiac phenotypes of *Ap2b1^m/m^* mutants | | | | | | | | | | | |
| --- | --- | --- | --- | --- | --- | --- | --- | --- | --- | --- | --- |
| Mutant ID | DORV | AVSD | Simple VSD | Right arch | Hypoplastic transverse arch | Hypo-plastic PA | MAPCA | Dual IVC | HTX | Normal Heart | Genotype |
| 2321-002-1 | 1 | 1 | 0 | 1 | 0 | 1 | 1 | 0 | 0 | 0 | *Ap2b1^m/m^* |
| 2321-004-NB | 0 | 0 | 1 | 0 | 1 | 0 | 0 | 0 | 0 | 0 | *Ap2b1^m/m^* |
| 2321-004-NC | 0 | 0 | 1 | 0 | 0 | 0 | 0 | 0 | 0 | 0 | *Ap2b1^m/m^* |
| 2321-006-MNE | 1 | 1 | 0 | 1 | 0 | 0 | 0 | 1 | 1 | 0 | *Ap2b1^m/m^* |
| 2321-006-NB | 1 | 0 | 0 | 0 | 0 | 0 | 0 | 0 | 0 | 0 | *Ap2b1^m/m^* |
| 2321-026-6 | 1 | 0 | 0 | 0 | 0 | 0 | 0 | 0 | 0 | 0 | *Ap2b1^m/m^* |
| 2321-026-9 | 0 | 0 | 0 | 0 | 0 | 0 | 0 | 0 | 0 | 1 | *Ap2b1^m/m^* |
| 2321-026-11 | 1 | 0 | 0 | 0 | 0 | 0 | 0 | 0 | 0 | 0 | *Ap2b1^m/m^* |
| 2321-104-NC | 0 | 0 | 0 | 0 | 0 | 0 | 0 | 0 | 0 | 1 | *Ap2b1^m/m^* |
| 2321-173-NB | 1 | 0 | 0 | 0 | 1 | 0 | 0 | 0 | 0 | 0 | *Ap2b1^m/m^* |
| 2321-264 | 0 | 0 | 0 | 0 | 0 | 0 | 0 | 0 | 0 | 1 | *Ap2b1^m/m^* |
| 2321-292 | 0 | 0 | 0 | 0 | 0 | 0 | 0 | 0 | 0 | 1 | *Ap2b1^m/m^* |
| 2321-364-2 | 1 | 0 | 0 | 0 | 0 | 0 | 0 | 0 | 0 | 0 | *Ap2b1^m/m^* |
| 2321-402-2 | 0 | 0 | 1 | 0 | 0 | 0 | 0 | 0 | 0 | 0 | *Ap2b1^m/m^* |
| Subtotal | 7 | 2 | 3 | 2 | 2 | 1 | 1 | 1 | 1 | 4 |  |
| AVSD: atrioventricular septal defect, DORV: double outlet right ventricle, HTX: heterotaxy, IVC: inferior vena cava, MAPCA: major aortopulmonary collateral arteries, PA: pulmonary artery, VSD: ventricular septal defect | | | | | | | | | | | |

| Supplemental Table 3. Extra-cardiac phenotypes of *Ap2b1^m/m^* mutants | | | | | | | | |
| --- | --- | --- | --- | --- | --- | --- | --- | --- |
| Mutant ID | Hydrops | Micro-  phthalmia | Cleft palate | Micrognathia | Hypoplastic Thymus | Syndactyly | Cystic kidney | Right lung isomerism |
| 2321-002-1 | 1 | 1 | 1 | 0 | 1 | 1 | 1 | 0 |
| 2321-004-NB | 0 | 0 | 0 | 1 | 0 | 0 | 0 | 0 |
| 2321-004-NC | 0 | 0 | 0 | 0 | 0 | 0 | 1 | 0 |
| 2321-006-MNE | 1 | 0 | 1 | 1 | 1 | 0 | 1 | 1 |
| 2321-006-NB | 0 | 0 | 0 | 1 | 1 | 0 | 0 | 0 |
| 2321-026-6 | 0 | NC | 0 | 0 | 0 | 1 | 0 | 0 |
| 2321-026-9 | 0 | NC | 0 | 0 | 0 | 0 | 0 | 0 |
| 2321-026-11 | 0 | NC | 0 | 0 | 0 | 0 | 0 | 0 |
| 2321-104-NC | 0 | 0 | 0 | 0 | 0 | 0 | NC | 0 |
| 2321-173-NB | 0 | 0 | 0 | 0 | 0 | 0 | NC | 0 |
| 2321-264 | 0 | 0 | 0 | 0 | 0 | 0 | 0 | 0 |
| 2321-292 | 0 | 0 | 0 | 0 | 0 | 0 | 0 | 0 |
| 2321-364-2 | 0 | 0 | 0 | 0 | 0 | 0 | NC | 0 |
| 2321-402-2 | 0 | 0 | 0 | 0 | 0 | 0 | NC | 0 |
| subtotal | 2 | 1 | 2 | 3 | 3 | 2 | 3 | 1 |
| NC: not check | | | | | | | | |

References

1. Li, Y., Klena, N.T., Gabriel, G.C., Liu, X., Kim, A.J., Lemke, K., Chen, Y., Chatterjee, B., Devine, W., Damerla, R.R., et al. (2015). Global genetic analysis in mice unveils central role for cilia in congenital heart disease. Nature *521*, 520-524. 10.1038/nature14269.

2. Guzman-Moreno, C., Zhang, P., Phillips, O.R., Block, M., Glennon, B.J., Holbrook, M., Weigand, L., Lo, C.W., and Lin, J.I. (2022). A Pipeline to Characterize Structural Heart Defects in the Fetal Mouse. J Vis Exp. 10.3791/64582.

3. Bahar, I., Atilgan, A.R., and Erman, B. (1997). Direct evaluation of thermal fluctuations in proteins using a single-parameter harmonic potential. Fold Des *2*, 173-181. 10.1016/S1359-0278(97)00024-2.

4. Case, D.A., Cheatham, T.E., 3rd, Darden, T., Gohlke, H., Luo, R., Merz, K.M., Jr., Onufriev, A., Simmerling, C., Wang, B., and Woods, R.J. (2005). The Amber biomolecular simulation programs. J Comput Chem *26*, 1668-1688. 10.1002/jcc.20290.

5. Salomon-Ferrer, R., Case, D.A., and Walker, R.C. (2013). An overview of the Amber biomolecular simulation package. WIREs Computational Molecular Science *3*, 198-210. <https://doi.org/10.1002/wcms.1121>.

6. Salomon-Ferrer, R., Gotz, A.W., Poole, D., Le Grand, S., and Walker, R.C. (2013). Routine Microsecond Molecular Dynamics Simulations with AMBER on GPUs. 2. Explicit Solvent Particle Mesh Ewald. J Chem Theory Comput *9*, 3878-3888. 10.1021/ct400314y.

7. Potapov, V., Cohen, M., and Schreiber, G. (2009). Assessing computational methods for predicting protein stability upon mutation: good on average but not in the details. Protein Eng Des Sel *22*, 553-560. 10.1093/protein/gzp030.

8. Capriotti, E., Fariselli, P., and Casadio, R. (2005). I-Mutant2.0: predicting stability changes upon mutation from the protein sequence or structure. Nucleic Acids Res *33*, W306-310. 10.1093/nar/gki375.

9. Hoang, T.T., Goldmuntz, E., Roberts, A.E., Chung, W.K., Kline, J.K., Deanfield, J.E., Giardini, A., Aleman, A., Gelb, B.D., Mac Neal, M., et al. (2018). The Congenital Heart Disease Genetic Network Study: Cohort description. PLoS One *13*, e0191319. 10.1371/journal.pone.0191319.

10. Arrigo, A.B., Zhu, W., Williams, K.A., Guzman-Moreno, C., Lo, C., and Lin, J.-H.I. (2023). Contribution of LRP1 in Human Congenital Heart Disease Correlates with Its Roles in the Outflow Tract and Atrioventricular Cushion Development. Genes *14*, 947.

11. Zhu, W., Williams, K., Young, C., Lin, J.H., Teekakirikul, P., and Lo, C.W. (2022). Rare and Common Variants Uncover the Role of the Atria in Coarctation of the Aorta. Genes (Basel) *13*. 10.3390/genes13040636.

12. Beecham, G.W., Bis, J.C., Martin, E.R., Choi, S.H., DeStefano, A.L., van Duijn, C.M., Fornage, M., Gabriel, S.B., Koboldt, D.C., Larson, D.E., et al. (2017). The Alzheimer's Disease Sequencing Project: Study design and sample selection. Neurol Genet *3*, e194. 10.1212/NXG.0000000000000194.

13. Chen, W., Liu, X., Li, W., Shen, H., Zeng, Z., Yin, K., Priest, J.R., and Zhou, Z. (2021). Single-cell transcriptomic landscape of cardiac neural crest cell derivatives during development. EMBO Rep *22*, e52389. 10.15252/embr.202152389.

14. Miao, Y., Tian, L., Martin, M., Paige, S.L., Galdos, F.X., Li, J., Klein, A., Zhang, H., Ma, N., Wei, Y., et al. (2020). Intrinsic Endocardial Defects Contribute to Hypoplastic Left Heart Syndrome. Cell Stem Cell *27*, 574-589 e578. 10.1016/j.stem.2020.07.015.

15. Liu, X., Chen, W., Li, W., Li, Y., Priest, J.R., Zhou, B., Wang, J., and Zhou, Z. (2019). Single-Cell RNA-Seq of the Developing Cardiac Outflow Tract Reveals Convergent Development of the Vascular Smooth Muscle Cells. Cell Rep *28*, 1346-1361 e1344. 10.1016/j.celrep.2019.06.092.

16. Lotto, J., Cullum, R., Drissler, S., Arostegui, M., Garside, V.C., Fuglerud, B.M., Clement-Ranney, M., Thakur, A., Underhill, T.M., and Hoodless, P.A. (2023). Cell diversity and plasticity during atrioventricular heart valve EMTs. Nat Commun *14*, 5567. 10.1038/s41467-023-41279-6.

17. Asp, M., Giacomello, S., Larsson, L., Wu, C., Furth, D., Qian, X., Wardell, E., Custodio, J., Reimegard, J., Salmen, F., et al. (2019). A Spatiotemporal Organ-Wide Gene Expression and Cell Atlas of the Developing Human Heart. Cell *179*, 1647-1660 e1619. 10.1016/j.cell.2019.11.025.

18. Hill, M.C., Kadow, Z.A., Long, H., Morikawa, Y., Martin, T.J., Birks, E.J., Campbell, K.S., Nerbonne, J., Lavine, K., Wadhwa, L., et al. (2022). Integrated multi-omic characterization of congenital heart disease. Nature *608*, 181-191. 10.1038/s41586-022-04989-3.
